# Role of nuclear pregnane X receptor in Cu-induced lipid metabolism and xenobiotic responses in largemouth bass (*Micropterus salmoides*)

**DOI:** 10.3389/fendo.2022.950985

**Published:** 2022-07-28

**Authors:** Hongyan Li, Wangbao Gong, Guangjun Wang, Ermeng Yu, Jingjing Tian, Yun Xia, Zhifei Li, Kai Zhang, Jun Xie

**Affiliations:** Key Laboratory of Tropical and Subtropical Fishery Resource Application and Cultivation, Pearl River Fisheries Research Institute, Chinese Academy of Fishery Sciences, Guangzhou, China

**Keywords:** PXR, lipid metabolism, Cu exposure, autophagy, apoptosis

## Abstract

The pregnane X receptor (PXR) is a master xenobiotic-sensing receptor in response to toxic byproducts, as well as a key regulator in intermediary lipid metabolism. Therefore, the present study was conducted to investigate the potential role of PXR in mediating the lipid dysregulation and xenobiotic responses under Cu-induced stress in largemouth bass (*Micropterus salmoides*). Four groups of largemouth bass (52.66 ± 0.03 g) were treated with control, Cu waterborne (9.44 μmol/L), Cu+RIF (Rifampicin, 100 mg/kg, PXR activator), and Cu+KET (Ketoconazole, 20 mg/kg, PXR inhibitor) for 48 h. Results showed that Cu exposure significantly elevated the plasma stress indicators and triggered antioxidant systems to counteract Cu-induced oxidative stress. Acute Cu exposure caused liver steatosis, as indicated by the significantly higher levels of plasma triglycerides (TG), lipid droplets, and mRNA levels of lipogenesis genes in the liver. Liver injuries were detected, as shown by hepatocyte vacuolization and severe apoptotic signals after Cu exposure. Importantly, Cu exposure significantly stimulated mRNA levels of PXR, suggesting the response of this regulator in the xenobiotic response. The pharmacological intervention of PXR by the agonist and antagonist significantly altered hepatic mRNA levels of PXR, implying that RIF and KET were effective agents of PXR in largemouth bass. Administration of RIF significantly exacerbated liver steatosis, and such alterations were dependent on the regulations on *pparγ* and *cd36* rather than *srebp1* signaling, which suggested that PXR-PPARγ might be another pathway for Cu-induced lipid deposition in fish. Whereas, KET administration showed reverse effects on lipid metabolism as indicated by the lower hepatic TG levels, suppressed mRNA levels of *pparγ* and *cd36*. Activation of PXR stimulated autophagy and inhibited apoptosis, leading to lower hepatic vacuolization; while inhibition of PXR showed higher apoptotic signals, inhibition of autophagic genes and stimulation of apoptotic genes. Taken together, PXR played a cytoprotective role in Cu-induced hepatotoxicity through regulations on autophagy and apoptosis. Overall, our data has demonstrated for the first time on the dual roles of PXR as a co-regulator in mediating xenobiotic responses and lipid metabolism in fish, which implying the potential of PXR as a therapy target for xenobiotics-induced lipid dysregulation and hepatotoxicity.

## Introduction

The aquaculture sector provides high-quality protein for human consumption and therefore plays a significant role in ensuring world food security. However, aquatic animals face with increasingly serious environmental stress induced by xenobiotics or chemicals such as microplastics, pesticides, and heavy metals from anthropogenic activities (1–3). Such environmental toxicants could result in stress and cause hepatotoxicity through several pathways including induction of oxidative stress, ER stress-induced apoptosis, or lipid dysregulation, therefore demonstrating disruptive effects on teleost fish (4, 5). However, far less information is available on the direct upstream regulators in the xenobiotic response in teleost fish.

The pregnane X receptor (PXR, also known as nuclear receptor NR1I2) and the constitutive androstane receptor (CAR, also known as nuclear receptor NR1I3) are ligand-activated nuclear receptors that mostly identified as xenosensors in response to toxic byproducts derived from endogenous metabolism and of exogenous chemicals (6, 7). In mammals, PXR and CAR mainly expressed in the liver and the intestine tissue, being the first defense line to inactivate and/or eliminate xenobiotics (8). However, CAR was lost from fish lineage in the evolutionary history, thus magnifying the potential role of PXR in xenobiotic responses in teleost fish (7, 9). Nowadays, PXR has been identified and cloned in teleost fish to explore its effects on detoxification pathways towards pharmaceuticals or pollutants, including zebrafish (*Danio rerio*), rainbow trout (*Oncorhynchus mykiss*), common carp (*Cyprinus carpio*), mosquito fish (*Gambusia affinis*), and *Mugilogobius abei* (10–14),. There were only limited studies elucidating the PXR-mediated xenobiotic response under metal exposure (15, 16). Thus, the direct involvement of PXR signaling in the metal-induced defense response has yet to be established.

In addition to recognize xenobiotic and chemical signals, PXR also contributes to the regulation of inflammation, cell proliferation, and energy metabolism including hepatic metabolism and gluconeogenesis (17, 18). Hepatic lipid homeostasis is a complex process maintained by balanced anabolism (lipogenesis) and catabolism (lipolysis and fatty acids β-oxidation). Several investigations have reported that PXR activation could inhibit lipid catabolism by provoking a downregulation in β-oxidation-related genes (19). Moreover, the activation of PXR was associated with increasement of transcript levels of peroxisome proliferators-activated receptor γ (PPARγ), which is a positive regulator of CD36 and a master regulator of adipogenesis (20). Therefore, PXR is an emerging new regulator of intermediary metabolism that connects the relationship between the sensing of chemical environment and the regulation of metabolic activities (21).

Moreover, accumulating evidences have indicated the impacts of environmental toxicants on lipid metabolism, in particular, on the incidence and progression of a worldwide occurring liver steatosis, non-alcoholic fatty liver disease (NAFLD) (22). Environmental pollutants such as heavy metals were reported to disturb lipid homeostasis in several kinds of teleost fish (23–25). Among these metals, copper (Cu) is an essential micronutrient required by all vertebrates, exerting physiological functions as a cofactor in the activity of a wide range of processes involved in cellular homeostasis and survival (26). However, exposure to excess Cu triggered lipid deposition in the liver of several fish, including zebrafish (*Danio rerio*), grass carp (*Ctenopharyngodon idella*), and *Synechogobius hasta* (24, 26, 27). The underlying molecular mechanisms of Cu-induced disorder in lipid homeostasis have been widely investigated in aquatic animals. Nevertheless, investigations on the link between the xenobiotic Cu exposure and downstream events, such as regulations on the related nuclear factors in lipid dysregulation, are lacking in teleost fish.

Largemouth bass (*Micropterus salmoides*) is one of the most important commercially cultured freshwater species in China. It also has been recognized as an indicator model for environmental toxicity assessment for its high-intensive and multi-regional rearing. To detect the potential role of PXR on the relationship between the sensing of xenobiotic metals and the regulation of lipid metabolism, pharmacological intervention was performed by using agonist and antagonist in the present study. Rifampicin (RIF) and ketoconazole (KET) were potent activator and inhibitor of PXR in mammals and some species of teleost fish, but their effects might be species-specific (1, 28). Previous reports had shown that RIF was an effective inducer on PXR-regulated CYP3A expression in largemouth bass (29). Therefore, acute Cu exposure experiments were carried out in largemouth bass, with the administration of RIF and KET as the activator and inhibitor of PXR, respectively. In the present study, plasma stress indicators, liver histologic and enzymatic assays, as well as responses to xenobiotic exposure, including liver molecular events were investigated.

## Materials and methods

### Fish and trial management

Largemouth bass were obtained and acclimatized for two weeks in ponds at the Pearl River Fisheries Research Institute, Guangzhou, China. Subsequently, two experiments were carried out in a statistical glass aquarium system. A 48-h acute Cu exposure test were firstly performed at an increasing concentration of Cu2^+^ at 15.74, 31.47, 47.21, 62.95, and 78.68 μmol/L in largemouth bass. Then the 48-hour median lethal concentration (LC50) was determined at 47.21 μmol/L, according to the prohibit analysis methods (30). Cu was supplemented by CuSO_4_·5H_2_O (C805353, Macklin, China) in distilled water to prepare stock solution. The amounts of CuSO_4_·5H_2_O were calculated according to the molar mass of Cu and total molar mass of CuSO_4_·5H_2_O, which ensured the actual concentration of Cu2^+^ at designed concentrations. Individual solutions used in the present study were dispensed by adding an appropriate volume of the primary stock solution to the dilution water.

Then, uniform-sized largemouth bass (body weight: 52.66 ± 0.03 g) were randomly assigned to four groups: Control, Cu waterborne (9.44 μmol/L, equal to 20% of 48-h LC_50_), Cu + RIF (Rifampicin, PXR activator, 100 mg/kg), and Cu + KET (Ketoconazole, 20 mg/kg, PXR inhibitor) for 48 h. Prior to the experiment, fish were anesthetized in diluted tricaine methane sulfonate solution (MS-222; Sigma, USA; 60 mg/L), weighted, and quickly intraperitoneally injected with the RIF or KET according to the body weight. Then, fish were immediately put into glass aquariums with prepared solutions containing Cu at a concentration of 0.06 mg/L for subsequent Cu exposure. All groups were set up as three replicates, with six fish per glass aquarium. Experiments in the present study were performed according to the guiding principles for the care and use of laboratory animals and were approved by Pearl River Fisheries Research Institute, Chinese Academy of Fishery Sciences (Approval ID: LAEC-PRFRI-2021-08-31).

### Sample collection

The fish were anesthetized with MS-222 at a concentration of 60 mg/L before sampling. Two fish for each glass aquarium were sacrificed at 48 h after acute Cu exposure. Blood was collected from the caudal vein of fish using syringes previously infiltrated with heparin sodium solution (0.2%, Sigma, USA). Then, plasma was separated after a centrifugation at 3000 g for 15 min. Parts of livers were removed and fixed in 4% formaldehyde for liver histochemical and histological observations. The rest parts of liver were immediately dissected on ice and frozen in liquid nitrogen, storing in the refrigerator at -80°C until subsequent analysis.

### Biochemical parameters and enzymatic analysis

Biochemical parameters including plasma glucose, triglycerides (TG), cortisol, and lactic acid levels were determined using commercial kits (A154-1-1, A110-1-1, H094, and A019-2-1; Jiancheng Bioengineering Institute, China). Liver tissues were accurately weighed and ground in sodium chloride buffer (0.9%) to prepare a 10% homogenate (w/v). Then, supernatants were obtained after centrifugation at 4°C for enzymatic analysis. Total antioxidant capacity (T-AOC), superoxide dismutase (SOD), catalase (CAT), and malondialdehyde (MDA) activities were determined using commercial kits according to the manufacturer’s recommendations (A015-2, A001-3-2, A007-1-1, and A003-1-2, Jiancheng Bioengineering Institute, China).

### Histological analysis

The fixed livers were dehydrated in ethanol gradients and embedded in paraffin. Crosssections were prepared by RM-2016 microtome at a thickness of 5 mm (Leica, Germany). Tissue sections were stained with hematoxylin and eosin (H&E), dehydrated and mounted. Observations were carried out under light microscopy (Olympus, Japan).

The previously fixed livers were sliced and embedded in TissueTek OCT compound (Sakura Finetek, Japan), rapidly frozen in liquid nitrogen-cooled isopentane, and cut into 5 μm sections with a cryostat. Sections were stained with neutral Oil Red O (Wako Pure Chemicals, Japan) to visualize the accumulation of fatty droplets in the liver, as described in previous studies (25). The TUNEL (Terminal-deoxynucleotidyl transferase-mediated nick end labeling) assay was carried out to observe the apoptotic signals induced by Cu exposure in the liver by detecting DNA fragmentation, and assays were performed according to methods described by Li etal. (5). Images were observed in in digital images from a Nikon Eclipse Ti-SR inverted microscope.

### Quantitative real-time polymerase chain reaction (qRT-PCR)

Total RNA was isolated from livers of largemouth bass using Trizol reagent (Invitrogen, USA). To assess the quality of RNA, agarose gel electrophoresis was performed to test the degradation and contamination of RNA. The RNA concentration and the A260/A280 ratio were checked using an Implen NanoPhotometer (Implen Inc.). Then, cDNA was synthesized using a PrimeScript^®^ RT reagent kit (TaKaRa, China) according to the manufacturer’s instructions. Quantitative RT-PCR was performed on a LightCycler 96 System (Roche, Switzerland) with SYBR^®^ Green I Master Mix (Roche, Germany). All reactions were performed in a 12 μl volume containing 4 μl of the cDNA template, 0.48 μl of the forward and reverse primer, 1.04 μl of ddH_2_O and 3 μl of LightCycle 480 SYBR Green 1 Master (Roche, Germany). Forty-five circles of PCR were performed, each consisting of heating at 95°C for 15 s for denaturing, and at 60°C for 10 s for annealing, and a third extension step at 72°C for 15 s. Melting curves were systematically monitored (with a gradient of 0.5°C/10 s from 55°C to 94°C) to confirm the specificity of the amplification reaction. Each PCR assay were set with replicates and negative controls were included. Relative expressions of genes evaluated by qPCR were calculated using a mathematical method based on the real-time PCR efficiencies, using a geometric mean of two reference genes (*β-actin* and *ef1α*) for the normalization. The primers used in the present study are shown in **Table S1**.

### Statistical analysis

All of the statistical analyses were performed with the SPSS (SPSS Inc., Chicago, USA) software. A Levene test were firstly conducted to verify the normality and homogeneity of variance, then a series of independent samples *t*-tests were conducted between the CON and Cu groups, Cu and Cu+RIF groups, as well as Cu and Cu+KET groups. Data were expressed as mean ± SEM (standard error of mean, n = 6), and the statistical significance level was considered as at *P* < 0.05. Significant differences between CON and Cu groups, Cu and Cu+RIF groups, as well as Cu and Cu+KET groups were indicated by “#”, “*”, and “+” (*P* < 0.05).

## Results

### Plasma stress indicators

**Figure 1:**
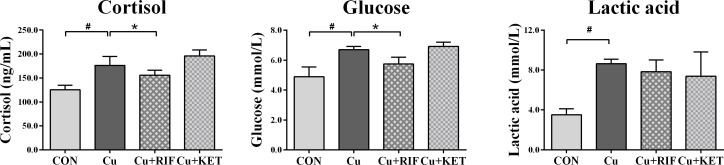
Plasma stress indicators in largemouth bass subjected to RIF or KET under Cu exposure (n = 6). Significant differences between CON and Cu groups, Cu and Cu+RIF groups, as well as Cu and Cu+KET groups were indicated by “#”, “*”, and “+” (*P* < 0.05).

The stress biomarkers in the plasma of largemouth bass subjected to RIF or KET under Cu exposure are presented in **Figure 1**. Results showed that Cu exposure significantly increased the cortisol, glucose and lactic acid levels in the plasma of largemouth bass (*P* < 0.05). Except for plasma lactic acid levels, administration of the PXR activator (RIF) significantly reduced levels of plasma stress indicators including cortisol and glucose (*P* < 0.05). While no significant alterations were observed on plasma parameters in groups of largemouth bass with KET administration (*P* > 0.05).

### Histological observations

**Figure 2:**
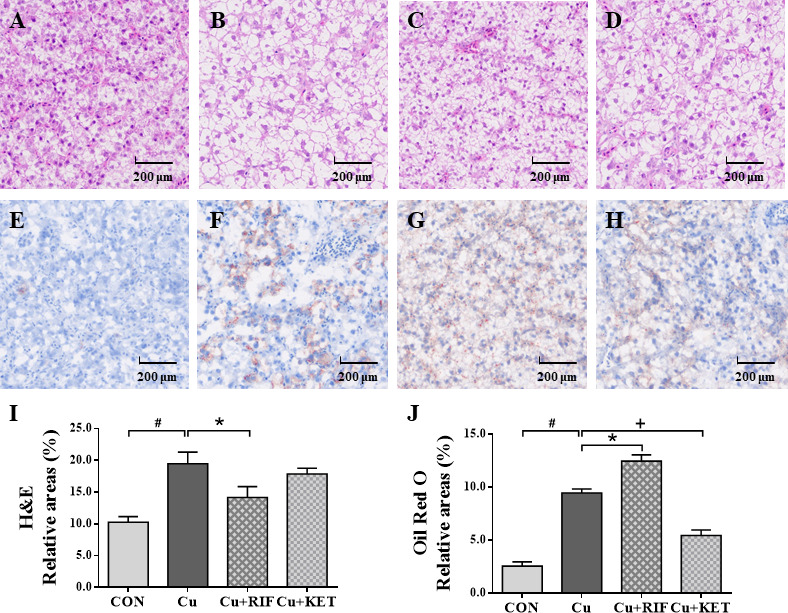
Liver histology (H&E) and histochemistry (Oil Red O) observations of largemouth bass subjected to RIF or KET under Cu exposure (n = 6). **(A, E)** Control; **(B, F)** Cu exposure; **(C, G)** Cu+RIF; **(D, H)** Cu+KET; **(I)** relative areas of hepatocyte vacuolization; **(J)** relative areas of lipid droplets. Photomicrographs magnification (× 200) and scale bar (200 μm). H&E: hematoxylin and eosin staining; Oil Red O: Oil red O staining. Lipid droplets was red-colored and nuclei was blue-colored. Data represent means ± SEM and are normalized to percentage of field area. Significant differences between CON and Cu groups, Cu and Cu+RIF groups, as well as Cu and Cu+KET groups were indicated by “#”, “*”, and “+” (*P* < 0.05).

The liver histology (H&E) and histochemistry (Oil Red O) observations of largemouth bass in the present study are shown in **Figures 2A**–**D** and **Figures 2E**–**H**, respectively. The structure of the liver in the control group displayed normal histology and polygonal cells, with a round nucleus with prominent nucleolus (**Figure 2A**). Acute Cu exposure resulted in vacuolization in hepatocytes of largemouth bass (**Figures 2B**–**D**), while RIF administration markedly reduced the portion of relative areas of vacuoles, as indicated by the quantificational results shown in **Figure 2I** (*P* < 0.05). Consistent with the H&E staining results, the Oil Red O staining showed that Cu exposure stimulated the appearance of lipid droplets in the liver of largemouth bass (**Figures 2F**–**H**). Moreover, significant differences were shown in Cu+RIF and Cu+KET groups as compared to the Cu exposure group individually, with higher relative areas of lipid droplets detected in the former and lower levels in the latter groups (**Figure 2J**; *P* < 0.05).

### PXR responses and lipid metabolism

**Figure 3:**
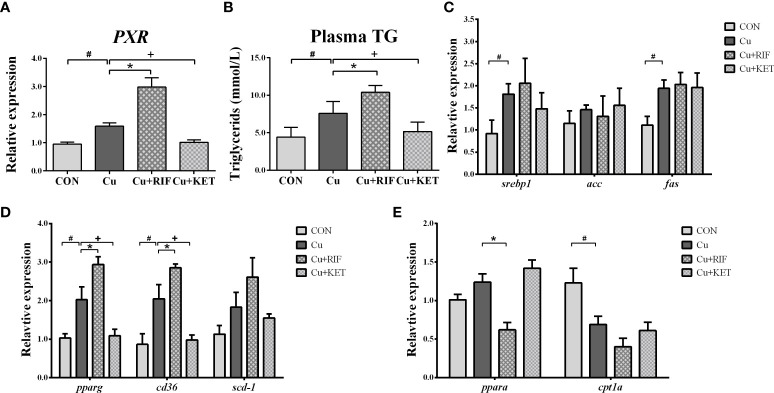
PXR responses and lipid metabolism related index in the liver of largemouth bass subjected to RIF or KET under Cu exposure (n = 6). **(A)** mRNA levels of PXR in the liver; **(B)** plasma triglycerides (TG); **(C)** hepatic mRNA levels of *srebp1*, *acc* and *fas*; **(D)** hepatic mRNA levels of *ppar*γ, *cd36* and *scd*-*1*; **(E)** hepatic mRNA levels of *pparα* and *cpt1a*. Significant differences between CON and Cu groups, Cu and Cu+RIF groups, as well as Cu and Cu+KET groups were indicated by “#”, “*”, and “+” (*P* < 0.05).

The response of the key regulator PXR was assessed by its transcriptional levels using qRT-PCR. The results showed that Cu exposure significantly stimulated PXR mRNA levels in the livers of largemouth bass (*P* < 0.05). Meanwhile, intraperitoneal administration of RIF and KET increased and decreased the mRNA levels of PXR, as compared to the Cu group (**Figure 3A**; *P* < 0.05).

In agreement with the Oil Red O staining results, TG levels were significantly elevated in the plasma of largemouth bass after Cu exposure (**Figure 3B**; *P* < 0.05). Compared to Cu group, significantly higher and lower levels of plasma TG were observed in the Cu+RIF and Cu+KET groups (*P* < 0.05). To detect the molecular events associated with lipid metabolism, mRNA levels involved in lipogenic genes and catabolic genes (fatty acids β oxidation) were determined and shown in **Figures 3C**–**E**. Acute Cu exposure stimulated the mRNA levels of *srebp1*, *fas*, *pparγ*, and *cd36* in the liver of largemouth bass (*P* < 0.05). On the contrary, Cu exposure significantly decreased the mRNA levels of *cpt1a* in the liver of largemouth bass (*P* < 0.05). With respect to the effects of administration of PXR activator and inhibitor, no alterations on the hepatic mRNA levels of *srebp1*, *acc*, and *fas* were found in either Cu+RIF or Cu+KET groups (*P* > 0.05). However, RIF administration significantly enhanced the mRNA levels of *pparγ*, *cd36* and suppressed the mRNA levels of *pparα* in the liver of largemouth bass after Cu exposure (*P* < 0.05). Along with this, the hepatic mRNA levels of *pparγ* and *cd36* were significantly decreased in largemouth bass subjected to intraperitoneal injection of KET after Cu exposure.

### Antioxidant systems

**Figure 4:**
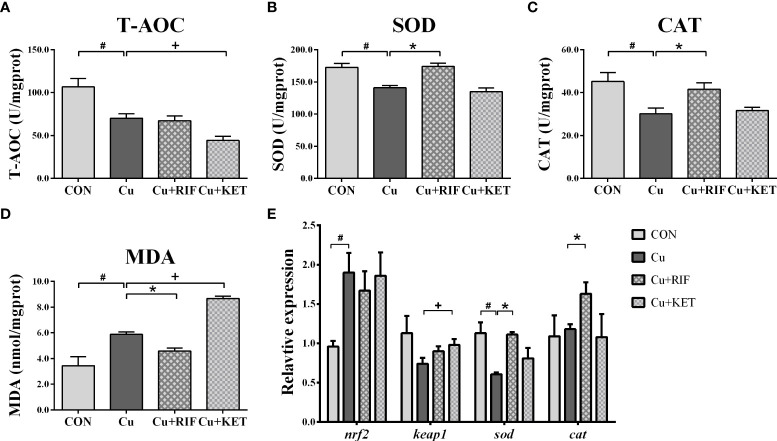
Enzyme activities and transcriptional levels of antioxidant systems in the liver of largemouth bass subjected to RIF or KET under Cu exposure (n = 6). **(A)** T-AOC: total antioxidant capacity; **(B)** SOD: superoxide dismutase; **(C)** CAT: catalase; **(D)** MDA: malondialdehyde; **(E)** hepatic mRNA levels of *nrf2*, *keap1*, *sod*, *cat1*. Significant differences between CON and Cu groups, Cu and Cu+RIF groups, as well as Cu and Cu+KET groups were indicated by “#”, “*”, and “+” (*P* < 0.05).

Acute Cu exposure induced significantly lower activities of T-AOC, SOD and CAT levels in the livers of largemouth bass (**Figures 4A**–**C**; *P* < 0.05). The MDA levels increased markedly after acute Cu exposure (**Figure 4D**; *P* < 0.05). As compared to the Cu group, the hepatic activities of SOD and CAT were significantly higher in the Cu+RIF group, while the T-AOC levels were significantly lower in the Cu+KET groups of largemouth bass (*P* < 0.05). Regarding the activities of MDA, RIF and KET administration significantly downregulated and upregulated the activities in the livers of largemouth bass, respectively (**Figure 4D**; *P* < 0.05).

For the expressions of genes associated with antioxidant signaling, acute Cu exposure significantly stimulated the mRNA levels of *nrf2*, and inhibited the mRNA levels of *sod* in the livers of largemouth bass (*P* < 0.05; **Figure 4E**). The administration of RIF induced higher mRNA levels of *sod* and *cat* in the livers of largemouth bass, and the administration of KET significantly elevated the mRNA levels of *keap1* in the livers of largemouth bass (*P* < 0.05).

### Autophagy and apoptosis

**Figure 5:**
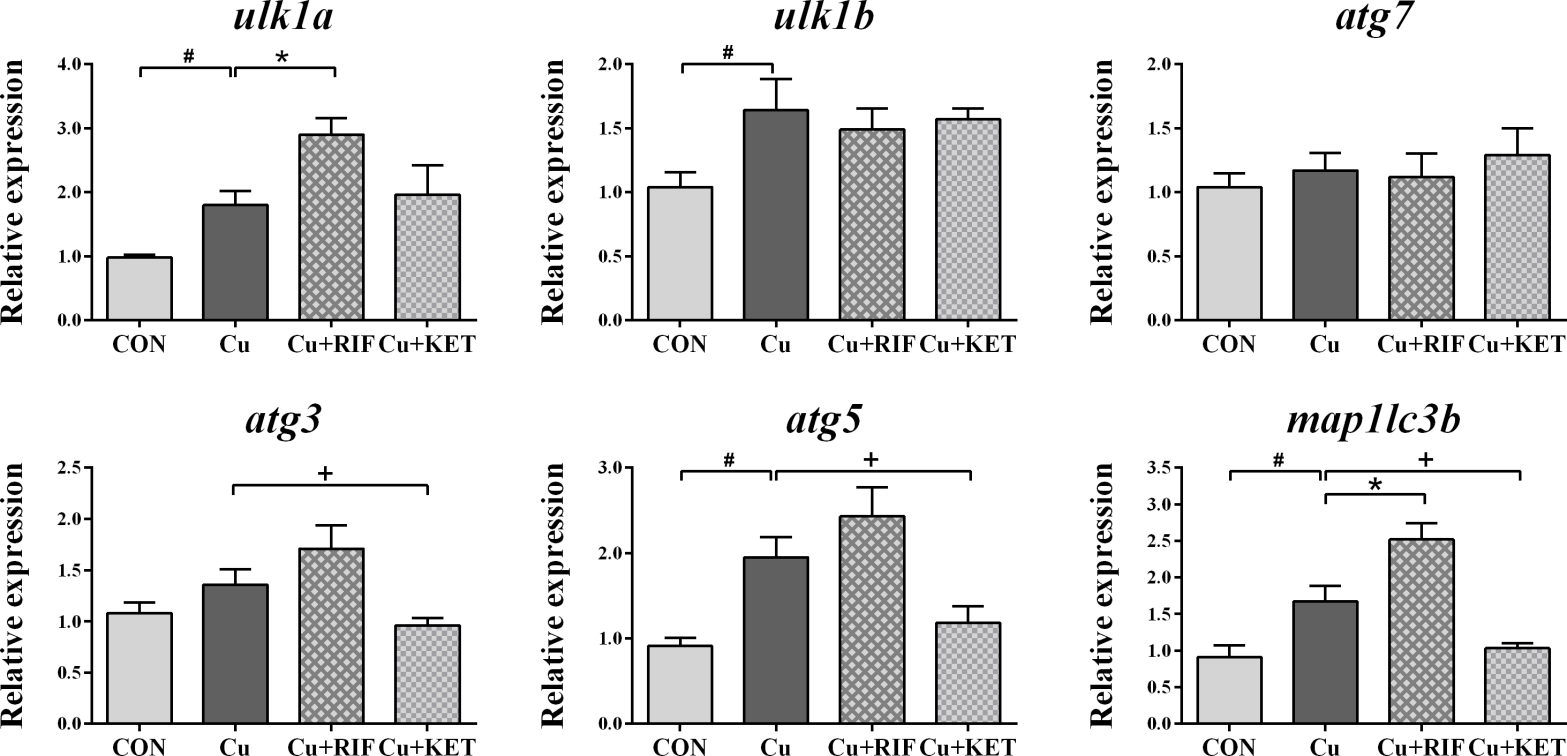
Hepatic mRNA levels of genes involved in autophagy in largemouth bass subjected to RIF or KET under Cu exposure (n = 6). Significant differences between CON and Cu groups, Cu and Cu+RIF groups, as well as Cu and Cu+KET groups were indicated by “#”, “*”, and “+” (*P* < 0.05).

The mRNA levels of genes involved in autophagy are presented in **Figure 5**. Acute Cu exposure significantly stimulated the transcriptional levels of *ulk1a*, *ulk1b*, *atg5*, and *map1lc3b* in the livers of largemouth bass (*P* < 0.05). Administration of RIF induced markedly higher mRNA levels of *ulk1a* and *map1lc3b* in the liver of largemouth bass, while KET significantly suppressed the mRNA levels of *atg3*, *atg5*, and *map1lc3b* (*P* < 0.05).

**Figure 6:**
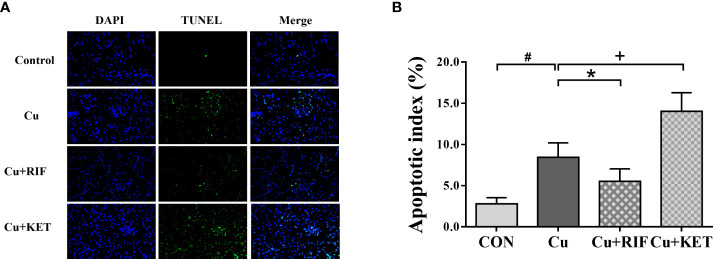
Representative DAPI and TUNEL double staining of liver in largemouth bass subjected to RIF or KET under Cu exposure (n = 6). TUNEL-positive cells were stained and indicated by bright green fluorescence indicating apoptotic cells, and normal nuclei appear in blue. **(A)** TUNEL staining sections; **(B)** Apoptotic index: the number of apoptotic nuclei/The number of observed nuclei × 100%. Significant differences between CON and Cu groups, Cu and Cu+RIF groups, as well as Cu and Cu+KET groups were indicated by “#”, “*”, and “+” (*P* < 0.05).

**Figure 7:**
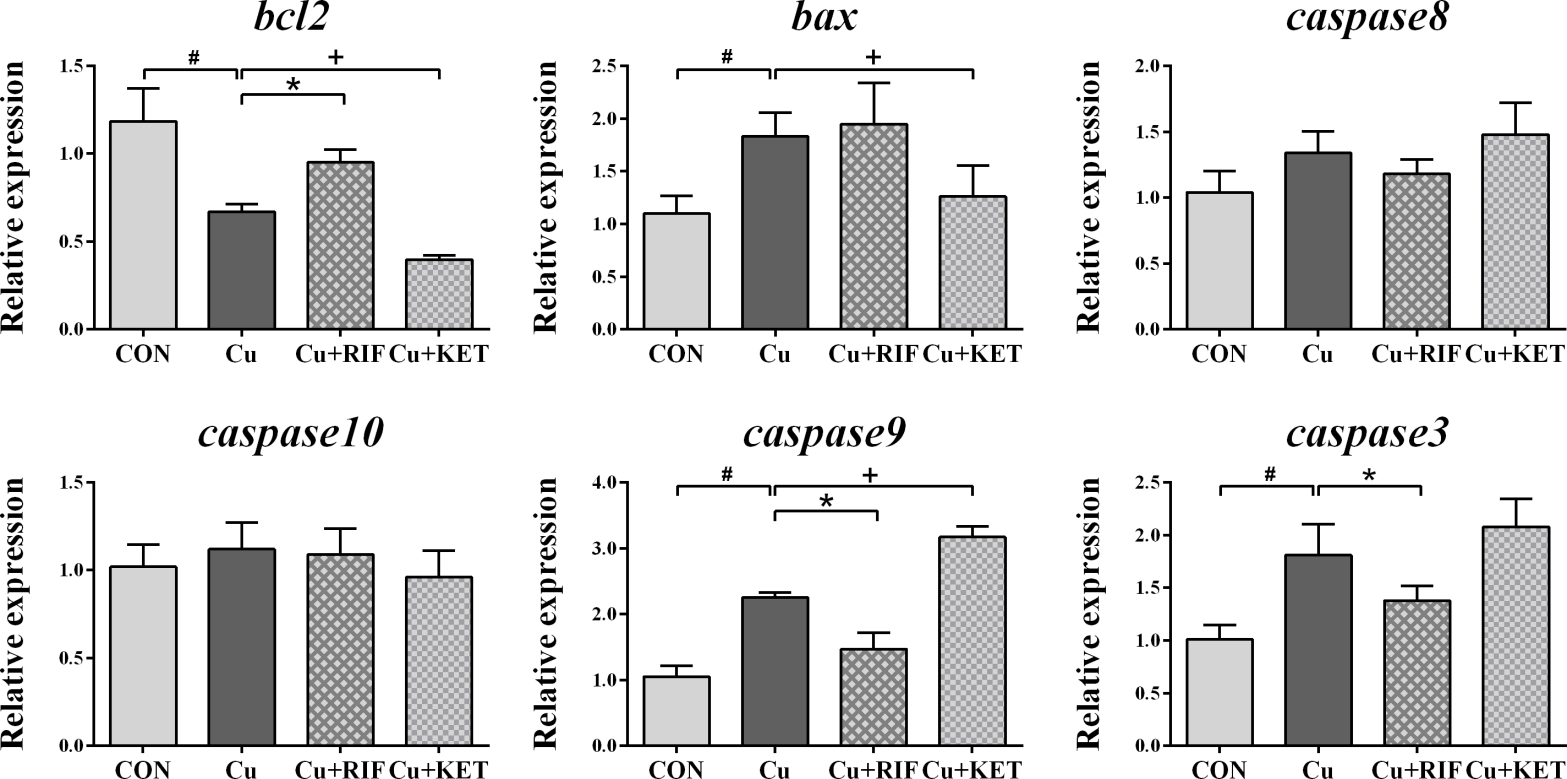
Hepatic mRNA levels of genes involved in apoptosis in largemouth bass subjected to RIF or KET under Cu exposure (n = 6). Significant differences between CON and Cu groups, Cu and Cu+RIF groups, as well as Cu and Cu+KET groups were indicated by “#”, “*”, and “+”(*P* < 0.05).

As shown by DAPI and TUNEL double fluorescence staining, rarely was apoptosis found in the CON group, while much many apoptotic signals were observed in all groups after Cu exposure (**Figure 6A**). The apoptosis rate in the Cu group was significantly higher than in the CON group (**Figure 6B**; *P* < 0.05). Meanwhile, administration of RIF and KET significantly elevated and decreased the apoptosis rate in the liver of largemouth bass after acute Cu exposure (*P* < 0.05). To better understand these effects, hepatic mRNA levels of genes involved in apoptotic processes were analyzed and presented in **Figure 7**. Acute Cu exposure significantly inhibited the mRNA levels of *bcl2*, while elevated the mRNA levels of *bax*, *caspase 9*, and *caspase 3* in the liver of largemouth bass (*P* < 0.05). Administration of RIF significantly increased the mRNA levels of *bcl2*, while decreased the mRNA levels of *caspase 9* and *caspase 3* (*P* < 0.05). On the contrary, significantly lower mRNA levels of *bcl2* and *bax*, as well as significantly higher of *caspase 9* were found in the largemouth bass of the Cu+KET groups (*P* < 0.05). No alterations were observed on the hepatic transcriptional levels of *caspase 8* and *caspase 10* in largemouth bass among all groups (*P* > 0.05).

## Discussion

### Cu-induced xenobiotic responses could be modified through pharmacological intervention of PXR

Stress is one of the most common responses in aquatic animals that are subjected to abnormal stimulation by environmental factors. Normally, plasma cortisol and glucose levels are considered as sensitive indicators of stress response in fish, which consistent of the primary and secondary stress systems (31). As a chemical byproduct of anaerobic respiration, LD increases when fish suffers stress. In the present study, 48-hour acute Cu exposure significantly elevated cortisol, glucose, and LD levels in the plasma, implying the occurrence of a stress response in largemouth bass after Cu exposure. Additionally, exposure to heavy metals such as Cu has been reported to result in oxidative stress and antioxidant response in various species of fish (25, 32). Nrf2-keap1 signaling is vital in cellular resistance to oxidants through regulating antioxidant and detoxifying genes (33). Acute exposure to Cu increased the gene expression of *nrf2*, suggesting a stimulation on the antioxidant pathways to counteract Cu-induced toxicity. The harmful effects of ROS generated from oxidative stress are balanced by activities of the non-enzymatic antioxidants and antioxidant enzymes including T-AOC, SOD, and CAT (34). However, Cu exposure finally induced a reduction on mRNA levels of *sod*, as well as on the enzymatic activities of T-AOC, SOD, and CAT in the liver of largemouth bass. The representative makers of peroxidation, MDA, were significantly higher in fish after Cu exposure. Higher portions of the relative areas of the vacuoles indicated that histological lesions were induced in the hepatocytes of largemouth bass after Cu exposure. Therefore, Cu exposure triggered antioxidant systems to fight against Cu-induced oxidative stress, but severe stress still caused hepatotoxicity in largemouth bass eventually.

To investigate the potential role of PXR in its response to the xenobiotic exposure, RIF and KET were applied as the activator and inhibitor in largemouth bass, respectively. Compared with Cu group, the markedly elevation and descent of mRNA levels of PXR in the livers suggested that RIF and KET were able to induce PXR activation and inhibition in largemouth bass. Thus, we further investigated the potential role of PXR in antioxidant responses after pharmacological intervention. Resistance to contaminants consists of detoxification and antioxidant pathways (35). The detoxification process is mainly regulated by downstream gens of PXR signaling, while the Nrf2-keap1 system activates target genes such as *sod* and *cat* to reduce the oxidative toxicity of environmental pollutants (36). In the present study, RIF administration stimulated the mRNA levels and enzymatic activities of SOD and CAT, as well as decreased the levels of MDA in the liver of largemouth bass after Cu exposure. Histological observations showed that the rate of hepatocyte vacuolation was significantly lower in the Cu+RIF group as compared to the Cu group, suggesting the cytoprotective role of PXR activation. The expression levels of the *nrf2* remained constant regardless of whether exposed to an agonist or antagonist. Nevertheless, the mRNA levels of *sod* and *cat* were significantly higher after subjected to RIF administration, as compared to the Cu group. Therefore, PXR showed positive roles in stress and antioxidant responses in largemouth bass, especially through pharmacological activation.

### PXR-PPARγ pathways might be one of mechanisms regulating Cu-induced hepatic steatosis

The peroxisome proliferator activated receptor γ (PPARγ) and the sterol-regulator element-binding protein 1 (SREBP1) are key nuclear factors involved in lipid metabolism. To date, PPARγ and SREBP1 have been shown to be vital intermediary factors in mediating the Cu-induced liver steatosis, by orchestrating the transcriptional levels of the lipogenesis-related enzymes (24, 37). In the present study, exposure to Cu caused significant stimulation of plasma TG levels, and hepatic mRNA levels of genes related to lipogenesis in largemouth bass, including *srebp1*, *fas*, *ppar*γ, and *cd36*. Consistently, significantly higher levels of lipid contents were retrieved in the liver of largemouth bass after Cu exposure, as indicated by the quantitative relative areas of lipid droplets according to Oil Red O staining. At the same time, the transcriptional levels of *cpt1a* were significantly lower after exposure to Cu, which represented the suppression of lipid catabolic pathways. Therefore, one of the mechanisms of Cu-caused hepatic steatosis was ascribed to the acceleration of the lipogenesis pathways and suppression of catabolic pathways in largemouth bass.

Nevertheless, the mechanisms as how Cu from xenobiotic exposure eventually alters hepatic lipid metabolism are still unclear. Therefore, investigations on the potential role of the xenobiotic stress sensor PXR in linking the xenobiotic responses and lipid metabolism emerged (38). The activation of PXR induced by environmental toxicants has been reported to be associated with liver steatosis (39). The present study showed that RIF and KET administration significantly increased and decreased the levels of plasma TG and lipid droplet levels in the liver of largemouth bass after Cu exposure respectively, suggesting the effective role of PXR in the regulation of Cu-induced lipogenesis. While taking the hepatic molecular events into account, we observed that the *srebp1* and the downstream gene *acc* and *fas* were constant whatever they were exposed to RIF or KET. Similarly, PXR was reported to induce lipogenesis in a SREBP1-independent manner in mammal models (17). Moreover, PXR activation was associated with up-regulation of PPARγ, a positive regulator of CD36 and a master regulator of adipogenesis (17). Concomitantly, RIF administration significantly enhanced the mRNA levels of *ppar*γ, as well as the downstream genes of *cd36* in the liver of largemouth bass after exposure. However, the administration of KET showed opposite effects, as refers to mRNA levels of *ppar*γ and *cd36*. Fatty acid β oxidation was also inhibited since mRNA levels of *pparα* were significantly lower in largemouth bass from the Cu+RIF group, which suggested the catabolism pathways also contributed to the lipid accumulation. In addition, recent studies have indicated that Cu exposure increased lipid deposition through oxidative stress-induced alteration of PPARγ pathways in yellow catfish (*P. fulvidraco*) (32). Therefore, our data indicated that pharmacological activation of PXR might induce hepatic steatosis through the stimulation of PXR-PPARγ and inhibition of fatty acid β oxidation by regulating PPARα pathways rather than alterations on SREBP1 signaling pathways.

### PXR protected against Cu-induced hepatoxicity *via* autophagy and apoptosis

Histological structure alterations represent lesions that fish suffered from hepatotoxicity due to heavy metal exposure. Fish have evolved systematic mechanisms and defense strategies to protect themselves from xenobiotic toxicity (5, 40). Autophagy is an important intracellular pathway for the degradation and recycling of cytosolic components, acting as either a survival or death safeguard mechanism depending on the environmental stress and cell type (41). Autophagy also mediated heavy metal-induced damage in tissues and organs (42). Ulk1, which belongs to a homologue of ATG1, is a key regulator in the initiation of autophagy by forming a complex with other autophagic proteins (43). In the present study, exposure to Cu caused significant induction on the mRNA levels of genes involved in autophagic process, including *ulk1a*, *ulk1b*, *atg5*, and *map1lc3b*. In accordance with this, the occurrence of autophagy acted as a defense strategy in gibel carp to combat metal-induced toxicology (25). In addition, apoptosis is a kinds of programmed cell death for maintaining cellular homoeostasis, which can be initiated under environmental stimuli and minimize the toxic effects of xenobiotics (4, 44). As compared to the control group, significant apoptotic signals shown by the TUNEL staining and significant higher apoptotic index were both observed in the Cu group. At molecular levels, significantly lower mRNA levels of the anti-apoptotic genes *bcl2* and higher levels of the pro-apoptotic genes *bax*, *caspase 9*, and *caspase 3* were observed after Cu exposure in largemouth bass. Taken the elevation of hepatocyte vacuolation into consideration, Cu exposure triggered the autophagy and apoptosis responses to alleviate hepatotoxicity in largemouth bass, but such regulations were still insufficient to countervail the occurrence of damage in the liver.

In addition, PXR may be a promising target for the prevention and treatment of liver disease based on its transcriptional regulation in inflammation, liver injury, and maintenance of homeostasis. PXR has also been shown to regulate liver autophagy and apoptosis pathways, thus having hepatoprotective effects in various liver injuries (44, 45). PXR-null mice were reported to have more severe liver damage and suppressed autophagy flow in mice (46). By using PXR-specific agonist and inhibitor to intervene in autophagy in mice with liver injuries, increasement of the autophagy indexes LC3-B and P62 were observed as a response to agonist pretreatment (46). In the present study, pharmacologic activation of PXR by RIF mitigated liver injuries, as shown by the lower levels of hepatocyte vacuolation in the H&E staining. Meanwhile, the mRNA levels of *ulk1a* and *maplc3b* were significantly triggered, which might verify the potential role of PXR on relieving liver damage *via* regulation on autophagic pathways. In addition to regulations on autophagic processes, PXR also promoted hepatocyte survival by upregulating the Bcl-xL and Bcl-2 anti-apoptotic proteins in rat and human (44). Constitutively activated PXR or pharmacologic activation of PXR by the agonist RIF in PXR-overexpressing cells protects human cells from deoxycholic acid-induced apoptosis (29). Our data revealed that administration of RIF significantly upregulated the mRNA levels of anti-apoptotic genes *bcl2* and downregulated the mRNA levels of pro-apoptotic genes *caspase9* and *caspase3.* Such anti-apoptotic effects were further confirmed by the lower rate of apoptotic index as shown by TUNLE staining. On the contrary, KET showed reverse effects, as lower mRNA levels of *bcl2* and higher levels of *caspase9*, eventually more severe apoptotic signals were observed in the liver of largemouth bass in the Cu+KET group. In conclusion, by intervening PXR with activators and inhibitors, our data demonstrated that PXR might play a protective role in Cu-induced liver injuries, and such positive effects were mainly involved in autophagy and apoptosis signaling pathways.

## Conclusion

Acute exposure to Cu caused stress, liver steatosis, and eventually liver injuries in largemouth bass. Such phenotypes were induced as to a combined involvements of antioxidant system, lipogenesis, autophagy, and apoptosis signaling pathways. For the role of PXR in Cu-induced lipid deposition and xenobiotic responses, our data indicated that RIF and KET were effective agonist and antagonist of PXR in largemouth bass. PXR promoted liver steatosis through regulations on lipogenesis and lipid catabolism in largemouth bass, but the lipogenesis enhancement was achieved majorly in a PPARγ-dependent manner rather than a SREBP1-dependent manner. Moreover, PXR showed cytoprotective role in mitigating Cu-induced liver injuries ascribed to its regulations on autophagy and its anti-apoptotic effects. Our findings provide the first evidence for the dual functions of PXR as a co-regulator in mediating metal-induced xenobiotic responses and intermediary lipid metabolism in fish, suggesting that PXR might be a promising target for therapy on stress-induced liver steatosis and hepatotoxicity through pharmacological intervention.

## Data availability statement

The original contributions presented in the study are included in the article/**Supplementary material**. Further inquiries can be directed to the corresponding author.

## Ethics statement

The animal study was reviewed and approved by Pearl River Fisheries Research Institute, Chinese Academy of Fishery Sciences.

## Author contributions

Funding Acquisition, Investigation, Methodology, Visualization, Writing - Manu Preparation, HL. Data Curation, Formal Analysis, WG and GW. Validation, EY and JT. Conceptualization, YX, ZL, and KZ. Supervision, Funding Acquisition, Writing - Review and Editing, JX. All authors contributed to the article and approved the submitted version.

## Funding

The work was financially supported by the Guangdong Basic and Applied Basic Research Foundation (2021A1515111077) and the National Key RandD Program of China (2019YFD0900302).

## Conflict of interest

The authors declare that they have no known competing financial interests or personal relationships that could have appeared to influence the work reported in this paper.

## Publisher’s note

All claims expressed in this article are solely those of the authors and do not necessarily represent those of their affiliated organizations, or those of the publisher, the editors and the reviewers. Any product that may be evaluated in this article, or claim that may be made by its manufacturer, is not guaranteed or endorsed by the publisher.
