# Supplementary material for: Role of nuclear pregnane X receptor in Cu-induced lipid metabolism and xenobiotic responses in largemouth bass (Micropterus salmoides)
Source: Front Endocrinol (Lausanne). 2022 Jul 28;13:950985. doi: 10.3389/fendo.2022.950985 (PMC9365941; doi:10.3389/fendo.2022.950985)
Supplement: Supplementary file 1 [file Table_1.docx]

| **Gene name** | **Forward (5′ - 3′)** | **Reverse (3′-5′ )** | **Size (bp)** |
| --- | --- | --- | --- |
| *ef1α* | TGCTGCTGGTGTTGGTGAGTT | TTCTGGCTGTAAGGGGGCTC | 147 |
| *β-actin* | ATCGCCGCACTGGTTGTTGAC | CCTGTTGGCTTTGGGGTTC | 336 |
| *pxr* | CAGGCGTGCCATAAAGAGGA | GGCTTGGCATTTACGGAAGC | 115 |
| *srebp1* | AGTCTGAGCTACAGCGACAAGG | TCATCACCAACAGGAGGTCACA | 127 |
| *acc* | ATCCCTCTTTGCCACTGTTG | GAGGTGATGTTGCTCGCATA | 121 |
| *fas* | GTCTGTGATGGCGAGGTGTC | CAGTCGGTATGAGGGTGTGG | 168 |
| *pparγ* | CCTGTGAGGGCTGTAAGGGTTT | TTGTTGCGGGACTTCTTGTGA | 103 |
| *cd36* | TCAGGAGACACAAGAGGGAAGA | TTGCCTGAACACCTTTACCGT | 124 |
| *scd-1* | TTCGCCTCCGCTTGAGATTT | GCATGGGTGGCATGTTGTTT | 141 |
| *pparα* | CCACCGCAATGGTCGATATG | TGCTGTTGATGGACTGGGAAA | 144 |
| *cpt1* | CATGGAAAGCCAGCCTTTAG | GAGCACCAGACACGCTAACA | 128 |
| *nrf2* | CAGACAGTTCCTTTGCAGGC | AGGGACAAAAGCTCCATCCA | 116 |
| *keap1* | CAGCATTACATGGCCGCATC | CTTCTCTGGGTCGTAAGACTCC | 86 |
| *sod* | TGGCAAGAACAAGAACCACA | CCTCTGATTTCTCCTGTCACC | 167 |
| *cat* | GTTCCCGTCCTTCATCCACT | CAGGCTCCAGAAGTCCCACA | 85 |
| *ulk1a* | ACTTCGCAATGTTGTCAGCG | ACAGTCCAACGACACTTCCC | 105 |
| *ulk1b* | CACTTCACCAGTTTGCCAGC | TTGGCTACACAGACCAGCAG | 108 |
| *atg7* | GGAGTTCGTTGCTCCTTACAT | CCAGCCACCGGGAAGTAAC | 141 |
| *atg3* | ATTGAGACTGTGGCGGAAGG | ATGACGGCCTGGACAAACTT | 83 |
| *atg5* | CCTGCTCCACTGCCATTCTAA | CTCTTGTGCTTGAGGGCGTC | 84 |
| *maplc3b* | GAAGCAGCGACATCAAACCG | TCGCGGCTGTATGAAAGCAA | 137 |
| *bcl2* | TGTGGGGCTACTTTTTGGCA | TTCGACTGCCACCCCAATAC | 57 |
| *bax* | ACTTTGGATTACCTGCGGGA | TGCCAGAAATCAGGAGCAGA | 133 |
| *caspase8* | GAGACAGACAGCAGACAACCA | TTCCATTTCAGCAAACACATC | 195 |
| *caspase10* | CAAACCACTCACAGCGTCTACAT | TGGTTGGTTGAGGACAGAGAGGG | 146 |
| *caspase9* | ATCCACGAGGGAGACAAAGAG | GCAACCGAGCACAAATAAGAG | 55 |
| *caspase3* | GCTTCATTCGTCTGTGTTC | CGAAAAAGTGATGTGAGGTA | 50 |

Supplementary Material

# Supplementary Table

**Table S1** Primers used in the present study.

Note: *ef1α:* elongation factor 1 alpha; *pxr*: pregnane X receptor; *srebp1*: sterol regulatory element binding protein 1; *acc*: acetyl-CoA carboxylase; *fas*: fatty acid synthase; *pparγ*: peroxisome proliferator-activated receptor *γ*; *scd-1*: stearoyl-CoA desaturase 1; *pparα*: peroxisome proliferator-activated receptor α; *cpt1a*: carnitine palmitoyl transferase 1 isoform a; *nrf2*: nuclear factor erythroid 2-related factor 2; *keap1*: kelch-like erythroid cell-derived protein-1; *sod*: superoxide dismutase; *cat*: catalase; *ulk1a*, *ulk1b*: autophagy protein 1 homologues; *atg7*: autophagy protein 7; *atg3*: autophagy protein 3; *atg5*: autophagy protein 5; *map1lc3b*: Microtubule-associated proteins 1A/1B light chain 3B; *bcl2*: B-cell lymphoma-2; *bax*: bcl-2-assoxicated x protein.
